# Effects of acute metaphedrone exposure on the development, behaviour, and DNA integrity of zebrafish (*Danio rerio*)

**DOI:** 10.1007/s11356-023-25233-z

**Published:** 2023-02-13

**Authors:** Ondina Ribeiro, Cláudia Ribeiro, Luís Félix, Isabel Gaivão, João Soares Carrola

**Affiliations:** 1 Centre for the Research and Technology of Agro-Environmental and Biological Sciences (CITAB), University of Trás-os-Montes and Alto Douro (UTAD), Quinta de Prados, 5000-801 Vila Real, Portugal; 2 TOXRUN—Toxicology Research Unit, University Institute of Health Sciences, CESPU, 4585-116 Gandra, CRL Portugal; 3 Interdisciplinary Centre of Marine and Environmental Research (CIIMAR), University of Porto, Edifício Do Terminal de Cruzeiros Do Porto de Leixões, Av. General Norton de Matos S/N, 4050-208 Matosinhos, Portugal; 4 Inov4Agro, Institute for Innovation, Capacity Building and Sustainability of Agri-Food Production, Vila Real, Portugal; 5 Veterinary and Animal Research Centre (CECAV), University of Trás-os-Montes and Alto Douro (UTAD), 5000-801 Vila Real, Portugal; 6 Department of Genetics and Biotechnology, University of Trás-os-Montes and Alto Douro (UTAD), 5000-801 Vila Real, Portugal; 7 Department of Biology and Environment (DeBA/ECVA), University of Trás-os-Montes and Alto Douro, CITAB, Vila Real, Portugal

**Keywords:** New psychoactive substances, Metaphedrone, Embryonic development, Behaviour, DNA damage, Zebrafish

## Abstract

The presence of new psychoactive substances (NPS), like metaphedrone (3-MMC), in aquatic environments raises concern about the potential negative effects on ichthyofauna. Therefore, the aim of this study was to evaluate the potential effects of 3-MMC on zebrafish embryonic development, behaviour, and DNA integrity. For that, embryos were exposed during 96 h post-fertilization to 3-MMC (0.1, 1, 10, and 100 µg/L). Overall, an increase in the eye area of zebrafish larvae was observed for the concentrations of 1 μg/L (increase of 24%) and 100 μg/L (increase of 25%) in comparison with the control group. Genetic damage was noted at the highest concentration (100 µg/L) with an increase of DNA damage (increase of 48%) and hyperactivity and disorganised swimming pattern characterised by an increase in speed (increase of 49%), total distance moved (increase of 53%), and absolute turn angle (increase of 48%) of zebrafish larvae. These findings pointed that, at environmental low levels, 3-MMC harmful effects are not expected to occur during critical development life stages of fish.

**Graphical Abstract:**

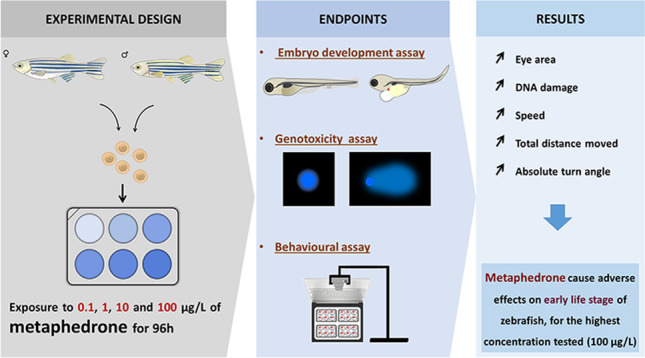

## Introduction

New psychoactive substances (NPS) were introduced as alternatives to traditional recreational drugs and are mainly synthetic, comprising distinct drug classes (e.g., cathinones, tryptamines, and opioids) (Shafi et al. 2020). Like other drugs of abuse or illicit, NPS are associated with several adverse health outcomes, such as substance use disorders, neurotoxicity, violence, and mortality. In 2021, the European Monitoring Centre of Drug and Drug Addition reported concerns regarding health and social risks caused by the manufacture and traffic of 3-methylmethcathinone, 3-MMC, also known as 2-(methylamino)-1-(3-methylphenyl)propan-1-one. The first appearance of 3-MMC was in Sweden in 2012 as a “legal drug” (Bäckberg et al. 2015) but rapidly spread all over Europe (Ferreira et al. 2019). The psychostimulant effects of 3-MMC were described as similar, although less potent and intense than those of 3,4-methylenedioxymethamphetamine and 4-methylmethcathinone (Sande 2016). Currently, 3-MMC is a controlled substance in several European countries (Bäckberg et al. 2015; Jamey et al. 2016; Silva et al. 2019). It has been described that 3-MMC has a stronger and more significant inhibition of the dopamine and norepinephrine transporters than for the serotonin transport and amphetamine-like stimulant properties (Luethi et al. 2018). Like other NPS, the consumption or direct disposal (excretion via urine) of 3-MMC can result in its presence in wastewaters and consequently in the aquatic ecosystems, raising concern about ecological risks (Bade et al. 2020b; Langa et al. 2021a). Synthetic cathinones are among the most consumed NPS, and their occurrence has been extensively reported in wastewaters for wastewater-based epidemiological studies (González-Mariño et al. 2016; Bade et al. 2020a; Langa et al. 2021a), but surface water levels are scarce or not reported. Nevertheless, environmental concentrations have been reported for some NPS and are usually within ng/L up to μg/L (Jin et al. 2022). Even at low concentrations, NPS have been shown to interfere with biochemical, physiological, and behavioural processes of nontarget species. For instance, exposure to cocaine and its metabolite caused changes in biochemical parameters of zebrafish embryos (Parolini et al. 2018a) and oxidative stress of daphnia (Parolini et al. 2018b). Exposure to ketamine caused physiological changes in the larvae of medaka fish (Liao et al. 2015). Nevertheless, in the grey literature, data about the potential ecotoxicological effects of 3-MMC is scarce (Shimshoni et al. 2015; Silva et al. 2019). With the increased consumption of this PAS, the assessment of its toxic effects has become important to provide scientific evidence on fish toxicity. Therefore, the need to understand the putative hazards and effects of 3-MMC on ecosystems is becoming increasingly important, particularly to fish.

Zebrafish as a model organism has risen in the latest years due to the relative transparency of the embryonic stages, high fecundity, small size, rapid developmental period (Meyers 2018), complex and robust behaviours (Wolman and Granato 2012) and well know genome (Howe et al. 2013). Thus, zebrafish’s early-life stages have been used to evaluate the toxicological effects of NPS (Kirla et al. 2021). For instance, MDMA increased spontaneous tail curling in zebrafish embryos, and the hatching rate was significantly faster (Barenys et al. 2022b). Also, recent toxicological evidence has shown that exposure to NPS impairs the DNA of zebrafish. The illicit drugs 25H-NBOH and 25H-NBOMe bind to the unclassical major groove of ctDNA, leading to conformational changes in the DNA structure (Barros et al. 2021). In addition, exposure to NPS has been shown to cause toxic effects on zebrafish behaviour. For example, Félix et al. (2017) demonstrated an increase in absolute turn angle in zebrafish larvae exposed to ketamine and found that 25C-NBOMe led to alterations in the motor response to a stimulus (Álvarez-Alarcón et al. 2021). Also, Kyzar et al. (2013) and Irons et al. (2010) showed that d-amphetamine and ketamine exposure evokes hyperactivity and anxiety in zebrafish. Contrarily, Barenys et al. (2022b) observed hypoactivity in zebrafish larvae exposed to MDMA.

Therefore, the main goal of the present study was to evaluate the potential effects of 3-MMC on embryonic development, genotoxic, and behavioural parameters during zebrafish early developmental stages.

## Materials and methods

### Ethics and welfare statement

The experimental procedures regarding maintenance and reproduction were performed in agreement with the European (Directive 2010/63/EU) and Portuguese legislations (Decreto Lei 113/2013) on animal experimentation and welfare. Fish were studied until 120 h post-fertilization (hpf); thus, no specific authorization is needed.

### Zebrafish maintenance, reproduction, and embryo collection

Adult AB wild-type zebrafish were kept at the University of Trás-os-Montes and Alto Douro (Vila Real, Portugal) in an open water system supplied with aerated, dechlorinated, charcoal-filtered, and UV-sterilised tap water from Vila Real City (pH 7.3–7.5), under controlled conditions of temperature (28 ± 1 °C) and photoperiod (14 h of light: 10 h dark), as described by Santos et al. (2020). Animals were fed twice a day with a standard diet for zebrafish (ZebraFeed, Sparos, Portugal).

The reproduction of adult zebrafish (ratio 1 female: 2 males) occurred in the early hours of the morning. After carefully collecting the embryos from the spawning tank, they were washed in E3 buffer (300 mM NaCl, 10.2 mM KCl, 20 mM CaCl_2_.2H_2_O, and 20 mM MgSO_4_.7H_2_O) and bleached with 0.5% chloramine-T to remove debris and clean the eggs.

### Experimental design

3-MMC (> 99% purity) was purchased from LGC Standards GmbH (Wesel, Germany). A stock solution at 1 g/L was prepared by dissolving the compound in methanol and stored at − 20 °C until use.

**Fig. 1:**
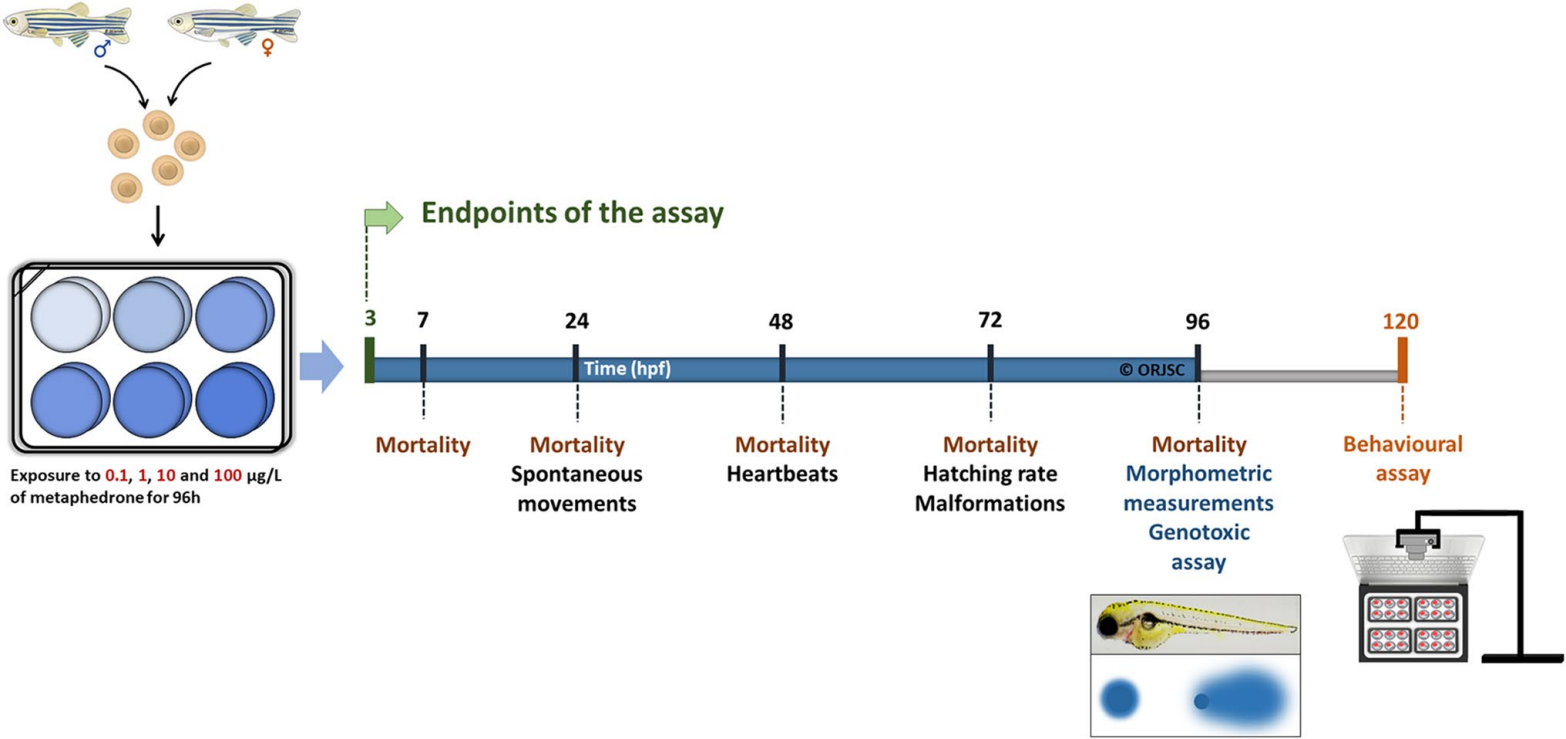
Scheme of the experimental design. After adult reproduction, 50 embryos per replicate with 3 hpf were exposed to 3-MMC at 0.1, 1, 10, and 100 µg/L or control solutions (E3 medium or methanol) until 96 h after exposure. During the time of exposure, embryonic development was evaluated, and at 96 hpf of exposure, morphometric measurements and genetic damage were assessed. At 120 hpf, the spontaneous and avoidance behaviour were evaluated

The experimental exposure of zebrafish for 96 h was conducted following the OECD 236 guideline (OECD 2013). Briefly, 5 replicates of 50 fertilised eggs (with approximately 3 hpf) were randomly distributed in each 6-well plate. For each replicate, 6 numbered papers (1–6) were used and placed inside the coat pocket and randomly removed to decide the order of the well to place the 50 eggs. Each well contained 5 mL of test solutions of 3-MMC at four different concentrations: 0.1, 1, 10, and 100 μg/L and control with E3 medium and with methanol at 0.01% were performed. Throughout the exposure test, the animals were maintained under the same controlled conditions of temperature (28 ± 1 °C) and photoperiod (14 h of light: 10 h of dark). The solutions were daily renewed, and dead embryos/larvae were counted and removed every day. The schematic view of experimental design is shown in Fig. 1.

### Zebrafish embryonic development parameters

Experiments were carried out according to Félix et al. (2014). For that, at 7, 24, 48, 72, and 96 hpf, zebrafish mortality was registered. The detachment of the tail and head were evaluated at 24 hpf. Spontaneous movements (24 hpf) and heartbeats (48 hpf) were quantified in 5 animals for 20 and 15 s, respectively. At 72 hpf, the hatching rate and the larval malformations were recorded. The morphometric measurements were measured at 96 hpf in 10 larvae randomly select from each replicate. For these, larvae were immobilised in methylcellulose (1%) and photographed using a stereomicroscope (Nikon SMZ800, Japan) coupled to a digital camera (Jenoptik) using ProgRes® CapturePro v2.8.8 software. The eye, yolk, oedema and head area, larva size, and tail curvature were measured using image analysis software. Also, organisms were examined for the presence of malformations.

### Genotoxicity assay

At 96 hpf, 10 larvae of each group were randomly collected for genetic damage evaluation. Briefly, with a clamp, the larvae in cold phosphate-buffered saline (PBS) without Ca^2+^ and Mg^2+^ (Merck KGaA, cat. no. P4417) were gently homogenised and then centrifuged (200 g for 3 min). The pellet was resuspended in cold PBS and centrifuged again. After resuspension in cold PBS, 300 µL of agarose with a low melting point (1%) (Merck KGaA, cat. no. A9414) were added to 30 µL of a solution, and then two drops (70 µL) were placed on a microscope slide pre-coated with agarose normal melting point (1%) (Merck KGaA, cat. no. A4718). A glass coverslip (18 × 18 mm) was placed on each drop to spread the mixture. After 5 min at 4 °C, the coverslip was removed. The slides were placed for 1 h in a Coplin-staining jar containing freshly prepared lysis buffer (2.5 M NaCl, 0.1 M EDTA, 10 mM Tris-base, 1% Triton X-100, pH 10, at 4 °C). After this time, the slides were incubated for 20 min in cold electrophoresis buffer (0.3 M NaOH, 1 mM EDTA, pH < 13.0 at 4 °C) to allow the DNA to unwind and 20 min in electrophoresis at 25 V (0.8 V/cm) and 300 mA. Lastly, the slides were neutralised in 1 × PBS and distilled water for 10 min, at 4 °C. To observe the comets, each gel was stained with 30 μL of 4′,6-diamidino-2-phenylindole (DAPI) and covered with a glass coverslip (22 × 22 mm) and observed on a fluorescence microscope, the Olympus BX41 (Olympus America, Inc., Hauppauge, NY, USA). Two slides were evaluated per replicate, and 100 aleatory nuclei per slide were visually scored. Each comet was classified based on the head integrity and tail length into five classes (Collins et al. 2008), namely zero (no tail) to four (almost all DNA in the tail). After that, the genetic damage index (GCI) was calculated and expressed as arbitrary units (AU) through the following equation: GDI = (*n*_0_ × 0) + (*n*_1_ × 1) + (*n*_2_ × 2) + (*n*_3_ × 3) + (*n*_4_ × 4), where *n* is the number of cells in each class analysed. As a result, the GDI could range from 0 (all cells without damage) to 400 AU (all cells with damage class 4).

### Larval behaviour assay

At 120 hpf, the behaviour experiment was performed in a room with a constant temperature (25 °C) but without light. Larvae presenting visible malformations were excluded from the behavioural analysis. To avoid shadows and blind spots in the footage, a central circular swimming area of agarose was stamped (27 mm diameter, 5 mm deep, and 4.5 mm thick). The 6-well plates were placed on top of the computer screen (4 groups of plates) upon a translucent cover (Leitz ColorClip 41,740,089) and used as a computer diffuser film to avoid moiré patterns. To minimise differences in experimental timing during the testing period, all tested groups were present in each plate. The video recording system used was previously described and implemented for zebrafish behavioural testing (Pelkowski et al. 2011). Following 2 min of acclimation, the spontaneous behaviour was evaluated for 10 min on a white background using the Real Fish Tracker software (version 0.4.0). The following spontaneous behaviour patterns were evaluated: speed, total distance moved, distance to the centre of the well, percentage of time in the upper and lower area of the well, absolute turn angle, and the percentage of active/inactive time of each larva.

### Statistical analysis

The statistical analysis was carried out using GraphPad Prism® software (version 8.00, GraphPad Software, San Diego, CA, USA). To analyse the normal distribution and homogeneity of the data, the Shapiro–Wilk and the Brown–Forsythe tests were performed, respectively. The different groups were compared by nonparametric independent samples Kruskal–Wallis’s test for non-normal distribution variables, and the results obtained were expressed as median and interquartile ranges. On the other hand, when the data had a normal distribution, they were analysed through one-way analysis of variance (ANOVA) followed by Tukey’s pairwise comparison tests, and the data were expressed as mean ± standard deviation.

## Results

### Effects of 3-MMC on embryonic development

**Fig. 2:**
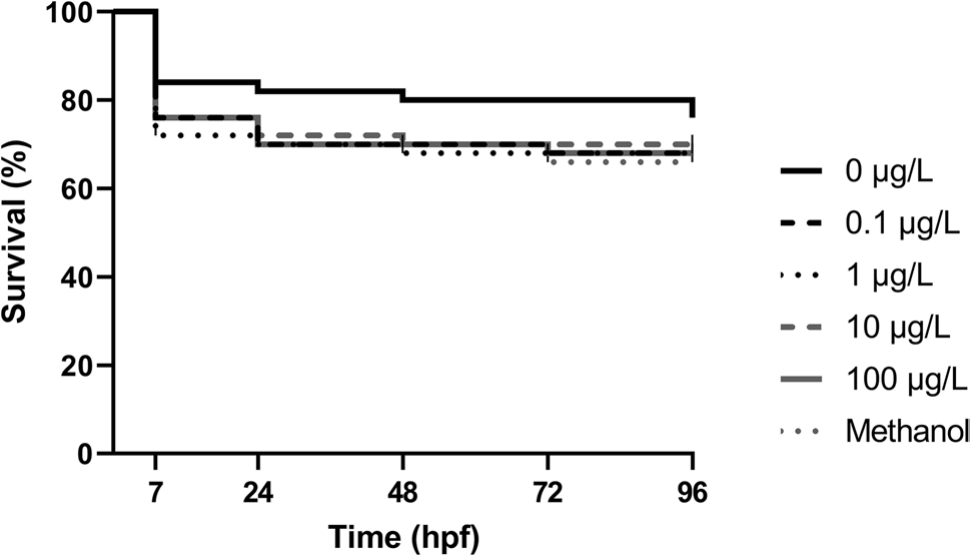
Survival of zebrafish embryos and larvae after exposure to 3-MMC until 96 hpf. Kaplan–Meier data are presented as a median of five independent replicates

Effects on embryonic development were assessed at 96 hpf. To determine the survival of zebrafish embryos and larvae, the mortality was recorded at 7, 24, 48, 72, and 96 hpf. The data observed in Fig. 2 suggest that 3-MMC do not induce statistically significant mortality in zebrafish at the different time points evaluated (7 hpf: *F*(5, 24) = 0.4827, *p* = 0.7871; 24 hpf: *F*(5, 24) = 0.2398, *p* = 0.9409; 48 hpf: *F*(5, 24) = 0.3399, *p* = 0.8836; 72 hpf: *F*(5, 24) = 0.3757, *p* = 0.8603; 96 hpf: *F*(5, 24) = 0.3955, *p* = 0.8470).

Embryonic development parameters were evaluated at 24 hpf (spontaneous movements, head detachment, and tail detachment), 48 hpf (heart rate), 72 hpf (hatching rate and malformations), and 96 hpf (morphometric measurements) (Table 1).

**Table 1:** Effects of 3-MMC exposure on zebrafish embryonic development at 24, 48, 72, and 96 hpf

Time (hpf)	Parameters	Concentrations (µg/L)						Statistical test	*P*-valu
		Control	0.1	e1	10	100	Methanol		
24	Spontaneous movements (mov/min)	3.9 (2.25–5.7)	4.2 (2.4–4.2)	4.2 (3–4.8)	2.4 (2.1–2.4)	2.4 (1.2–3)	3.6 (1.8–4.2)	*X*^2^(5) = 7.420	0.1912
	Head detachment (%)	ND	ND	ND	ND	ND	ND	N/A	N/A
	Tail detachment (%)	ND	ND	ND	ND	ND	ND	N/A	N/A
48	Heart rate (bpm)	178.72 ± 8.13	171.2 ± 4.63	168 ± 3.44	171.68 ± 3.33	169.12 ± 8.19	173.28 ± 3.60	*F*(5, 24) = 2.262	0.0807
72	Hatching rate (%)	ND	ND	ND	ND	ND	ND	N/A	N/A
	Malformations (%)	8.57 (3.23–12.50)	5.13 (3.23–6.25)	5.56 (0–7.69)	0 (0–2.63)	3.23 (0–3.57)	4.32 (3.11–7.63)	*X*^2^(5) = 6.321	0.2762
96	Size larvae (mm)	3.34 (3.10–3.54)^ab^	3.14 (3.14–3.39)^a^	3.51 (3.46–3.80)^ab^	3.69 (3.29–3.71)^ab^	3.65 (3.60–3.78)^b^	3.34 (3.30–3.39)^ab^	*X*^2^(5) = 15.10	0.0099
	Eye area (mm^2^)	0.07 (0.06–0.08)^a^	0.08 (0.08–0.09)^ab^	0.09 (0.09–0.09)^b^	0.08 (0.08–0.09)^ab^	0.09 (0.09–0.09)^b^	0.09 (0.09–0.09)^ab^	*X*^2^(5) = 16.45	0.0057
	Yolk area (mm^2^)	0.30 (0.30–0.41)	0.29 (0.24–0.34)	0.27 (0.27–0.35)	0.26 (0.26–0.30)	0.28 (0.26–0.29)	0.28 (0.26–0.30)	*X*^2^(5) = 6.286	0.2793
	Oedema area (mm^2^)	0.05 (0.03–0.15)	0.06 (0.04–0.06)	0.05 (0.03–0.06)	0.03 (0.03–0.09)	0.04 (0.04–0.07)	0.05 (0.04–0.07)	*X*^2^(5) = 1.060	0.9576
	Head area (mm^2^)	0.28 (0.27–0.32)	0.29 (0.29–0.35)	0.32 (0.30–0.35)	0.31 (0.30–0.31)	0.34 (0.32–0.34)	0.33 (0.33–0.33)	*X*^2^(5) = 8.025	0.1548
	Tail curvature (°)	175.78 (175.78–177.43)^ab^	163.14 (144.84–163.14)^a^	175.99 (175.99–177.41)^b^	170.21 (170.21–177.02)^ab^	175.70 (172.20–177.13)^ab^	168. 38 (168.38–170.30)^ab^	*X*^2^(5) = 16.05	0.0067
	Malformations (%)	0.20 (0.14–0.20)	0.63 (0.63–0.83)	0.38 (0.17–0.38)	0.25 (0.14–0.57)	0.29 (0.29–0.43)	0.38 (0.29–0.43)	*X*^2^(5) = 10.14	0.0713

Data are expressed as mean ± SD for parametric data distribution or median and interquartile range for nonparametric data, of five independent replicates. Statistical analysis was performed using one-way ANOVA followed by Tukey’s multiple-comparison test or Kruskal–Wallis. Different letters indicate significant differences between groups (*p*-value < 0.05). *ND*, not detected; *N/A*, not applicable.

**Fig. 3:**
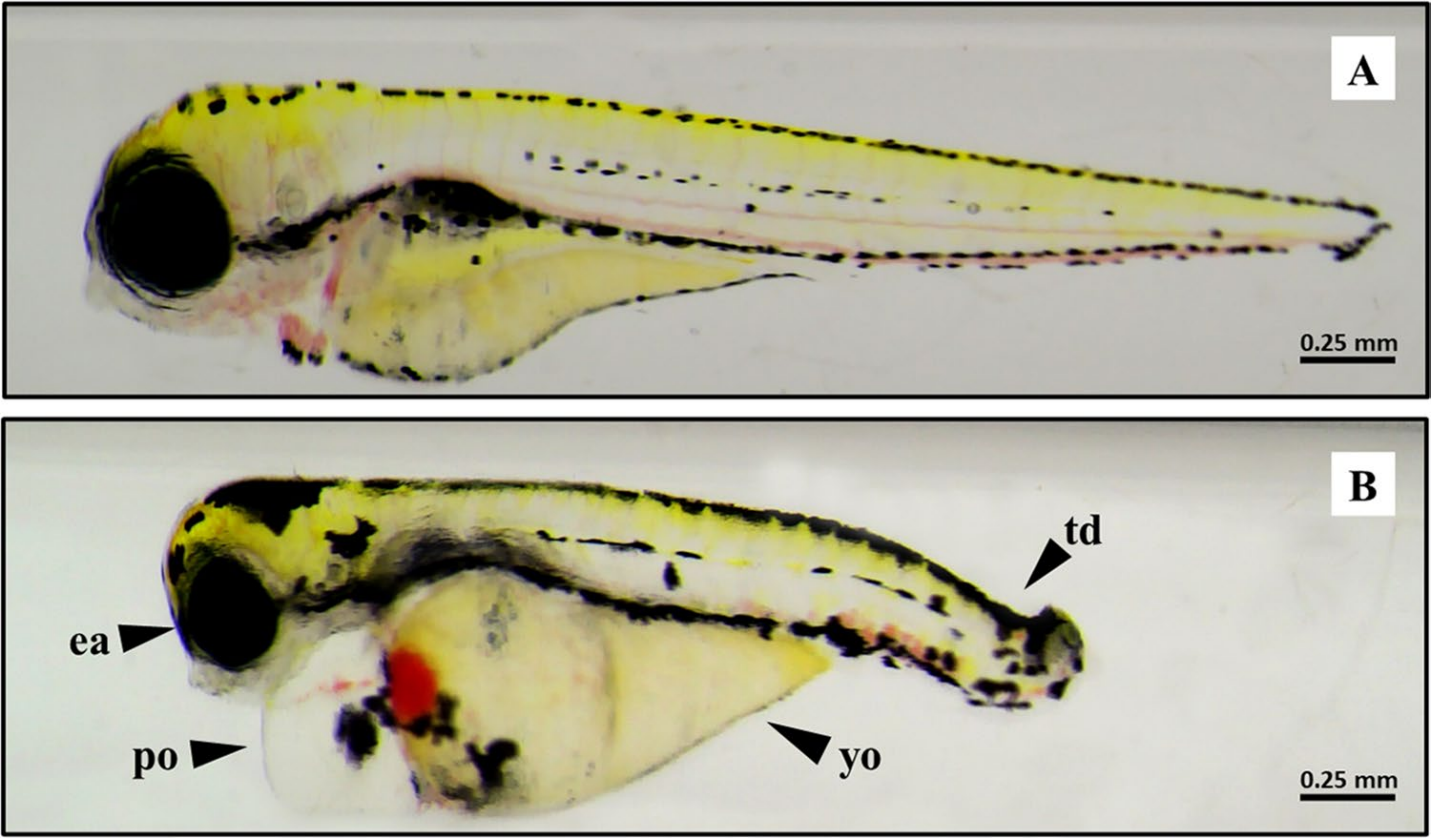
Representative optical images of zebrafish larvae at 96 hpf after exposure to 3-MMC. **A** Larvae exposed to control concentration; **B** larvae exposed to 100 μg/L of 3-MMC showed tail deformation (td), eye area reduction (ea), yolk sac oedema (yo), and pericardium oedema (po)

At 96 hpf, size larvae, eye area, yolk area, oedema area, head area, and tail curvature (morphometric measurements) were analysed in 10 arbitrarily selected larvae from each well. In addition, malformations in zebrafish larvae were registered. The main malformations observed at 96 hpf were deformations in the tail, eye area reduction, yolk sac oedema, and pericardium oedema (Fig. 3).

No significant effects were observed between the control and all tested concentrations for the selected parameters. However, an increase in the eye area was observed in the zebrafish larvae exposed to 1 (*p* = 0.0477) and 100 μg/L (*p* = 0.0160) compared to the control. Also, a decrease in size of larvae was observed for the concentration of 0.1 μg/L when compared to 100 μg/L, and an increase in tail curvature for the concentration of 0.1 compared to 1 μg/L. Despite these alterations, no significant effects in these parameters were observed when compared to the control.

### DNA damage after exposure to 3-MMC

**Fig. 4:**
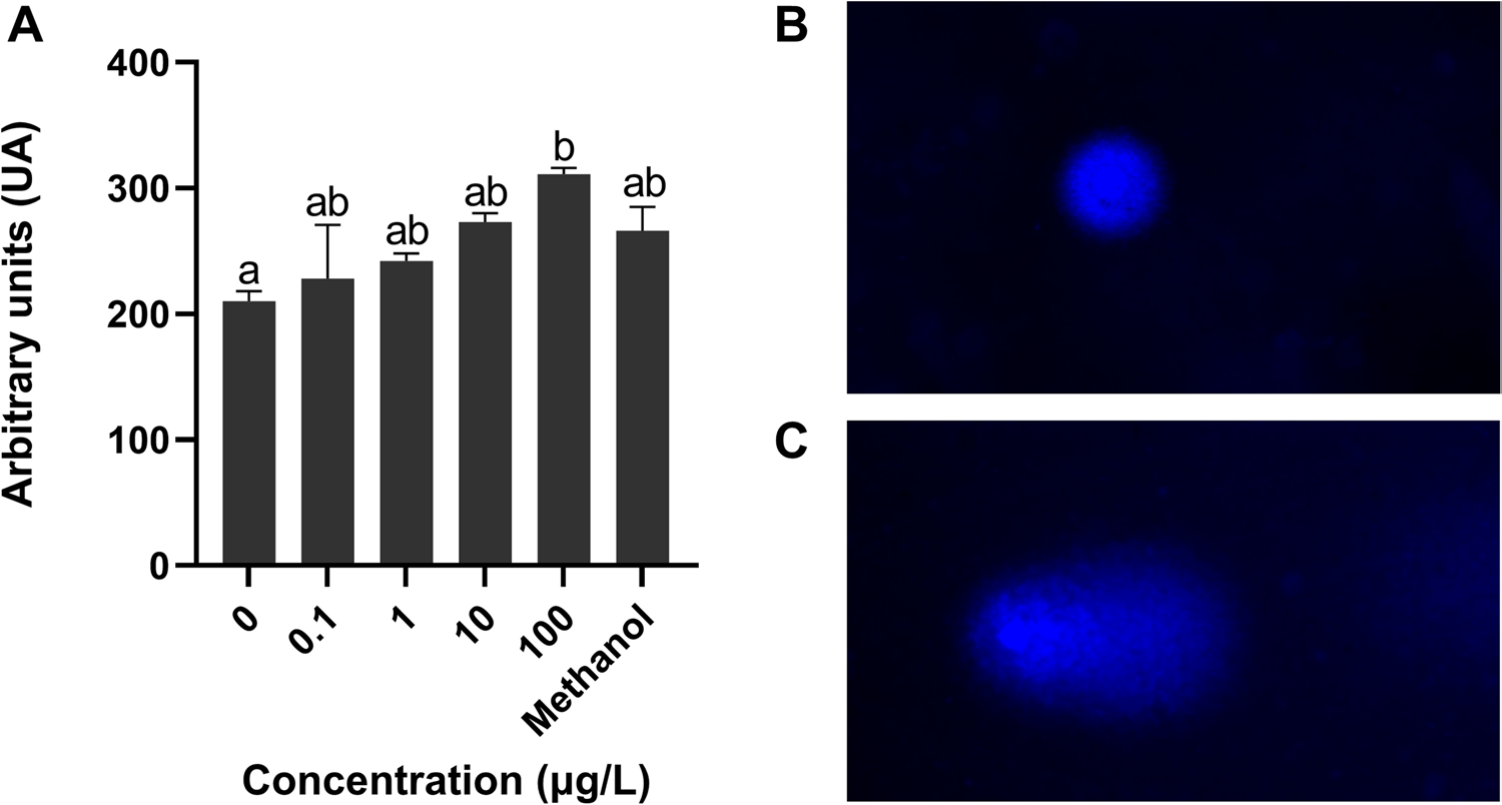
Genetic damage of zebrafish larvae exposed to 3-MMC at 96 hpf (**A**). Nonparametric data are presented as a median and interquartile range, of five independent replicates. Statistical analysis was performed using the Kruskal–Wallis test. “*” indicate significant differences between groups (*p*-value < 0.05). Comet images of larvae cells showing different migration patterns, according to the levels of DNA damage, class 0 (**B**), and class 4 (**C**)

The genetic damage was evaluated by the alkaline comet assay in zebrafish larvae exposed to different concentrations of 3-MMC after 96 hpf. A dose-dependent increase in the DNA damage was observed (*X*^2^(5) = 19.73, *p* = 0.0014); however, only at the highest concentration (100 μg/L), a significant increase (*p* = 0.0009) was observed compared to the control (Fig. 4).

### Evaluation of zebrafish behavioural parameters after exposure to 3-MMC

**Fig. 5:**
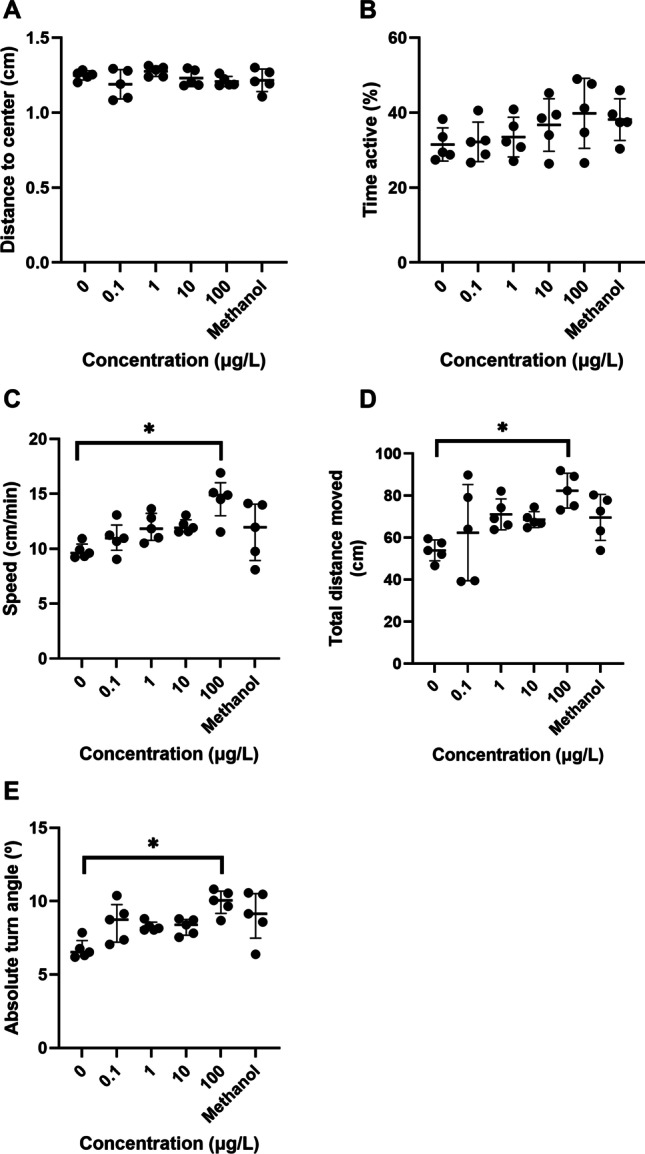
Spontaneous behaviour of zebrafish larvae exposed to 3-MMC at 120 hpf. **A** Speed, **B** total distance moved, **C** distance to the centre of the well, **D** percentage of the time active, and **E** absolute turn angle. Values were expressed as mean ± SD (graphs B, C, and D) or median and interquartile range (graphs A and E) of five independent replicates. Statistical analysis was performed using one-way ANOVA followed by Tukey’s multiple-comparison test or Kruskal–Wallis. “*” indicate significant differences between groups (*p*-value < 0.05)

No significant effects were observed in the distance to the centre of the well (*F*(5, 24) = 1.321, *p* = 0.2887; Fig. 5A) and the time activity of the zebrafish larvae (*F*(5, 24) = 1.437, *p* = 0.2470; Fig. 5B) at 120 hpf. On the other hand, exposure to 3-MMC induced an increment of the speed (*X*^2^(5) = 14.16, *p* = 0.0146) in the concentration of 100 μg/L (*p* = 0.0174) compared to the control group (Fig. 5C). Similarly, larvae presented an increase in the total distance moved (*F*(5, 24) = 3.340, *p* = 0.0198; Fig. 5D) and the absolute turn angle (*X*^2^(5) = 14.80, *p* = 0.0113; Fig. 5E) when exposed to the highest concentration compared with the group control (*p* = 0.0321 and 0.0079, respectively).

## Discussion

This study considered a variety of variables (morphophysiological, swimming behaviour, and genotoxicity) to give a thorough understanding of the potential toxicological impact of 3-MMC during critical stages of the early development of zebrafish. The occurrence and levels of various synthetic cathinones, including 3-MMC, have been reported in wastewaters (Salgueiro-González et al. 2022; Bade et al. 2022); nevertheless, surface water levels are scarce (Langa et al. 2021b). Therefore, different concentrations of 3-MMC were chosen according to possible environmental concentrations of other similar NPS to and at higher sublethal concentrations to evaluate the toxic effects of 3-MMC on zebrafish embryos and larvae. To our knowledge, this study is the first report that provides data on the toxicological effects of 3-MMC in zebrafish.

### Effects of 3-MMC on embryonic development

The early life stages of zebrafish are sensitive to the adverse effects of drugs and their exposure can lead to problems during embryo development. Our data showed that exposure to 3-MMC causes morphometric changes in zebrafish embryonic development as an increase in the eye area at 1 and 100 μg/L. No other morphometric changes were observed.

Similar to our results, no morphometric changes in embryonic development and cumulative mortality were observed in zebrafish exposed to venlafaxine, a chiral antidepressant, at *s* concentrations ranging from 0.3 to 3000 μg/L (Ribeiro et al. 2022). Also, the exposure to pyrovalerone, a synthetic cathinone, did not cause mortality in embryos and larvae zebrafish (Souders et al. 2019). In contrast, Kalichak et al. (2016) showed that the exposure of zebrafish to fluoxetine, a classical serotonin reuptake inhibitor antidepressant, decreased the heart rate and length of larvae at the highest concentration (99 mg/L). Also, the exposure to MDMA increased spontaneous tail coiling (966.25 and 9662.5 µg/L) in zebrafish embryos, and the hatching rate was significantly faster at all MDMA-tested concentrations (966.25, 4831.25, and 9662.5 µg/L) (Barenys et al. 2022b). In these studies, the fluoxetine and MDMA concentrations were much higher than selected concentrations of 3-MMC for this study. Additionally, another study done by Kalichak et al. (2016) showed that the exposure of zebrafish to fluoxetine (0.009 to 99 µg/L), diazepam (0.008 to 88 µg/L), and risperidone (0.00033 to 33 µg/L) affected the initial development of these animals. These psychoactive drugs increased the mortality rate and heart frequency and decreased larvae length. Fluoxetine, diazepam, and risperidone modulate the neurotransmitter systems and their receptors and, thus, can have detrimental effects on the central and peripheral systems in initial stages of zebrafish development. These compounds may show different effects on embryonic development from those observed in exposure to 3-MMC, as they to different classes of psychoactive substances with distinct activities than 3-MMC (synthetic cathinone).

### DNA damage after exposure to 3-MMC

DNA damage can occur spontaneously or under the influence of environmental factors during normal DNA metabolism due to spontaneous changes in the chemical structure of bases (Alak et al. 2021). DNA damage, which includes base modifications, abasic sites, and strand breaks, is one major consequence of oxidative stress. In fact, when the ROS overproduction exceeds the capacity of the cell machinery to scavenge and detoxify the reactive intermediates, oxidative stress induces damage to vital cellular structures, like nucleic acids, which impairs DNA integrity (Gonzalez-Hunt et al. 2018; Da Silva et al. 2014).

Our results showed that exposure to 3-MMC (100 µg/L) caused a significant increase in DNA damage in larvae cells, confirming the compound’s ability to induce primary genetic lesions. This increase in genetic damage has been reported in freshwater organisms exposed to amphetamine (Parolini et al. 2016a) and benzoylecgonine (Parolini et al. 2018b; Cadet et al. 2007; El-Tawil et al. 2011) related to the overproduction of ROS. In addition, in fish and bivalves it was observed that the mechanism of action of MDMA, amphetamine, methamphetamine and ketamine seems to unbalance the oxidative state of organisms, leading to an increase in ROS levels (Magni et al. 2016; Parolini et al. 2016b; Félix et al. 2018, 2016; Liao et al. 2018, 2015), and consequently induction of genetic damage.

Parolini et al. (2016a) observed that exposure of zebra mussels to 0.5 and 5 µg/L of amphetamine led to a significant increase in DNA fragmentation and primary genetic lesions in zebra mussel haemocytes. Therefore, similar mechanisms may be related to 3-MMC genotoxic potential; however, more studies are needed to corroborate the evidence of the damage to the genetic damage via oxidative stress or other molecular mechanisms.

### Evaluation of zebrafish behavioural parameters after exposure to 3-MMC

The capability for normal swimming behaviour is crucial for fish growth, reproduction, and survival in the natural environment (Liao et al. 2015). Behavioural changes can be caused by embryonic developmental or neurological effects and is an important endpoint for sublethal toxicological evaluation in zebrafish (Sloman and McNeil 2012).

In this work, we observed that exposure to 3-MMC can negatively affect the swimming activity of the larvae. However, the toxic effects of 3-MMC on behaviour may be independent of embryonic development, as no noted toxic effects of 3-MMC were observed throughout embryonic development in zebrafish. Exposure of larvae to 3-MMC induced alterations in speed and total distance moved, with an increase of these parameters for the higher concentration (100 µg/L). So, our results suggest that exposure of zebrafish larvae to 100 µg/L of 3-MMC induces hyperactivity and hyperlocomotion. Exposure to *d*-amphetamine and ketamine exposure evokes hyperactivity and anxiety in zebrafish and medaka fish, respectively (Kyzar et al. 2013; Irons et al. 2010; Liao et al. 2015). Amphetamine-like substances have some well-known behavioural effects, such as an increase in locomotor activity and hyperactivity in exposed animals in response to a rapid release of dopamine and serotonin in the nucleus accumbens (Kehr et al. 2011; Kyzar et al. 2013; Baumann et al. 2007). 3-MMC may also act in the neurotransmitter’s transporters (Luethi et al. 2018), which can lead to behavioural disruption in the underlying circuits that control the development of locomotor function (Barenys et al. 2022b, 2022a).

Also, an increase in the absolute turn angle (the amount of turning irrespective of direction) was observed in larvae exposed to 100 µg/L. Liao et al. (2015) showed that medaka fish larvae exposed from the early blastula stage until 7 days to methamphetamine (59.7 and 597 µg/L) appeared to move in a clockwise direction due to an increased absolute turn angle. Additionally, quinpirole-treated zebrafish exhibited increased absolute turn angle (Nabinger et al. 2021). The increase in the average of this behavioural parameter may suggest a disorganised locomotor pattern of the larvae with the presence of elevated erratic movements, which may be a response to increased fear or anxiety or alterations in morphological development (Budick and O'Malley 2000). At the beginning of the behavioural assay, larvae with visible morphological alterations were discarded; however, it is possible that the increase in the eye area observed is related to a rise in motor activity, which may indicate possible neurobehavioral disorders after exposure to 3-MMC. Like rodents and humans, the main neuroendocrine regulation of the integrated stress response in zebrafish is mediated by cortisol. In addition, cortisol regulates a diverse range of systems, including behaviour, and is an indicator of stress (Steenbergen et al. 2011). In a study performed with healthy male humans, it was found that a dose of 200 mg of 4-MMC led to an increase in plasma cortisol concentrations. These authors assume that the serotonergic effects of 4-MMC can lead to stimulation of the hypothalamic–pituitary–adrenal axis (Papaseit et al. 2020), which translates into an increase in plasma cortisol concentrations as seen with other psychoactive substances (Strajhar et al. 2019). Similarly, zebrafish possess a hypothalamic–pituitary–adrenal axis that is comparable to humans, with cortisol as a primary mediator of physiological response to stress (Fonseka et al. 2016). Thus, exposure of zebrafish to 3-MMC may lead to behavioural changes through increased cortisol levels in the hypothalamic–pituitary–adrenal axis. However, further studies are needed to verify whether 3-MMC has effects on these mechanisms with implications for zebrafish behaviour.

## Conclusions

This is the first study that reports the effects of 3-MMC on the development of zebrafish on different biomarkers of toxicity, such as morphometric, genotoxicity, and swimming behaviour. In summary, our results demonstrate that the 3-MMC can negatively affect zebrafish larvae, causing adverse impacts in several parameters studied, namely embryonic development, DNA damage, and behaviour. The exposure of zebrafish to 3-MMC shows an increase in the eye area and in genetic damage, which can affect DNA integrity with an increase in the chance of malignant transformation, among other health effects/diseases. Additionally, zebrafish larvae exhibit hyperactivity and disorganised swimming pattern, related to the increase in speed, total distance moved, and absolute turn angle of zebrafish larvae. Nevertheless, these results were only observed for the highest concentrations (100 µg/L) of 3-MMC. The present study shows the importance to do more research to understand the toxic effects of 3-MMC, which may explain the results observed since information on the toxicity of this compound is scarce. In conclusion, additional studies are required, mainly about 3-MMC environmental concentrations since harmful effects cannot be discarded at lower concentrations due to potential interactions between the environmental chemicals and natural stressors.

## Data Availability

All data generated and analysed are included in the published article, supplementary material and upon reasonable request to the corresponding author João Carrola.
